# Developing a molecular picture of soil organic matter–mineral interactions by quantifying organo–mineral binding

**DOI:** 10.1038/s41467-017-00407-9

**Published:** 2017-08-30

**Authors:** C. J. Newcomb, N. P. Qafoku, J. W. Grate, V. L. Bailey, J. J. De Yoreo

**Affiliations:** 1 Pacific Northwest National Laboratory, 902 Battelle Boulevard, Richland, Washington 99354 USA; 2 Department of Materials Science and Engineering, University of Washington, 302 Roberts Hall, Seattle, Washington 98195 USA

## Abstract

Long residence times of soil organic matter have been attributed to reactive mineral surface sites that sorb organic species and cause inaccessibility due to physical isolation and chemical stabilization at the organic–mineral interface. Instrumentation for probing this interface is limited. As a result, much of the micron- and molecular-scale knowledge about organic–mineral interactions remains largely qualitative. Here we report the use of force spectroscopy to directly measure the binding between organic ligands with known chemical functionalities and soil minerals in aqueous environments. By systematically studying the role of organic functional group chemistry with model minerals, we demonstrate that chemistry of both the organic ligand and mineral contribute to values of binding free energy and that changes in pH and ionic strength produce significant differences in binding energies. These direct measurements of molecular binding provide mechanistic insights into organo–mineral interactions, which could potentially inform land-carbon models that explicitly include mineral-bound C pools.

## Abstract (web summary)

Most molecular scale knowledge on soil organo–mineral interactions remains qualitative due to instrument limitations. Here, the authors use force spectroscopy to directly measure free binding energy between organic ligands and minerals and find that both chemistry and environmental conditions affect binding.

## Introduction

From a thermodynamic perspective, soil organic matter (SOM) should readily turnover, however, due to complex interactions among plants, microbes, and minerals, it can reside in soils for months to millennia^1, 2^. Currently, it is well accepted that the persistence and decomposition of organic compounds in soil is interconnected with the physical environment, organic–mineral interactions, and both the local biotic and abiotic factors. Long residence times of SOM are commonly attributed to sorption of organic species to mineral surfaces, which provide reactive sites for physical and chemical stabilization that prevent SOM degradation by enzymes and microbes^1–4^. Additionally, soil geochemistry has recently been demonstrated to be a robust predictor of soil carbon storage, emphasizing the role of minerals in carbon turnover^5, 6^.

To date, most experimental data that probe organic–mineral interactions have been empirical and provide qualitative trends, making it difficult to directly incorporate the findings into mineral-C pools in land-carbon models^4, 7^. Quantifying binding at the organic matter–mineral interface would enable researchers to directly compare binding strengths of specific SOM molecules with known chemistry, which could provide insight into the stabilization of C pools. Current techniques such as batch adsorption^8^ and calorimetry experiments^9^ provide indirect methods for probing SOM–mineral interactions, while spectroscopic techniques and molecular and surface complexation modeling provide molecular level details^10^. Researchers have gained significant knowledge using these techniques, however, there are some disadvantages. Data from adsorption experiments lack face specificity, representing instead an average of all faces, edges, and fracture surfaces. In addition, depending on the chemistry and concentration of the ligand, molecules may interact with one another, potentially competing for organic–mineral binding. Calorimetry can be challenging to interpret, and Fourier transform infrared spectrometry (FTIR) is not amenable to probing aqueous conditions with low surface area minerals. Fourier transform ion cyclotron resonance has also begun to make large strides in identifying the chemistry of SOM associated with minerals and soil pores^11, 12^, however because it is based on mass spectrometry, absolute quantification of the results is challenging. Understanding binding at the molecular level could provide important information for deducing the importance of specific SOM chemistry or mineralogy of a soil in carbon stability.

Here we report a proof-of-concept approach using dynamic force spectroscopy (DFS), an in situ technique that provides a quantitative molecular scale measurement of molecular binding at a mineral surface. DFS measurements can be designed to systematically probe increasing levels of molecular complexity, including multivalency^13^ and ligand mixtures, in well-defined environmental conditions while avoiding confounding parameters such as variable surface area and unknown distributions of distinct mineral faces. Additionally, DFS provides numerical values for the energy landscape of the bond^14^, which can provide mechanistic insights into interactions that occur at larger length scales.

DFS uses a functionalized atomic force microscopy (AFM) cantilever, which is allowed to interact with a surface so that a bond between the functionalized tip and the surface is formed. The cantilever is then pulled from the surface until the bond is broken. This approach is also used in chemical force microscopy (CFM, also referred to chemical force spectroscopy), a technique similar to DFS, but that only probes rupture forces at a single pulling velocity. In contrast, DFS probes rupture forces over a large range of pulling velocities. As a result, it explores bond rupture dynamics in two fundamental regimes: a near-equilibrium regime at low pulling rates and a kinetic, non-equilibrium regime at high pulling rates^14, 15^. In the near-equilibrium regime, bond breaking is stochastic, being driven by thermal fluctuations, and thus provides information about the ratio of the inherent binding and unbinding rates. In the non-equilibrium regime, bond breaking becomes deterministic and one can extract valuable information about bond kinetics. By fitting the entire range of data using established analyses, one can extract quantitative values for the intrinsic rate where the bond transitions from a bound to unbound state (*k*_o_), the lifetime of the bond (*x*_t_), and the equilibrium free energy of binding (Δ*G*_b_)^16–18^.

In this work, we utilize DFS to probe organic–mineral interactions in a quantitative manner that allows us to make comparisons between specific functional groups and mineral types with varying environmental conditions. In particular, DFS can probe interactions that other techniques have yet to explore in the soil science community. The data provides unambiguous face-specific measurements that can begin to address the complexity of soils using a bottom-up approach. For example, the role of ligand chemistry, local environment, or specific mineral faces can be probed directly. The data presented here focus on simple functional groups as a starting point and demonstrate that environmental factors can have a strong influence on binding. The approach is readily extended to more complex, realistic biomolecules and systems characterized by ongoing biological activity.

## Results

### Establishing a model system for organic–mineral interactions

**Fig. 1:**
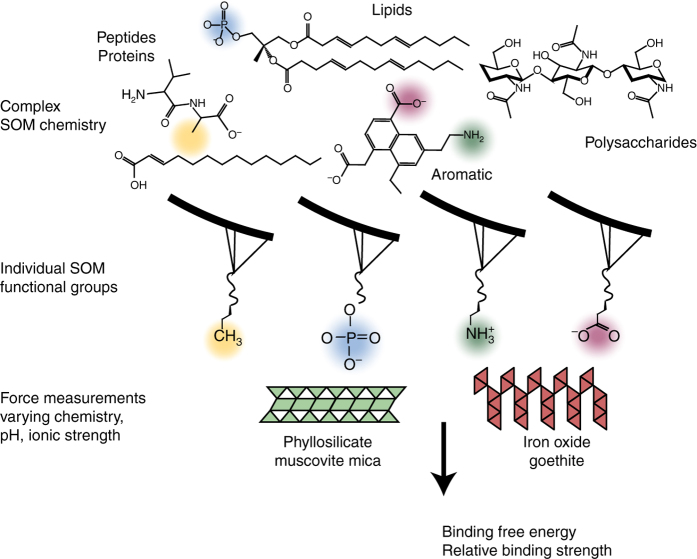
Schematic of the experimental setup. Common chemical functional groups from soil organic matter (SOM) were chosen to represent a model for interaction between organics and two model minerals: muscovite and goethite. Performing force measurements between these model organics and minerals enable us to quantitatively evaluate the binding energies

**Fig. 2:**
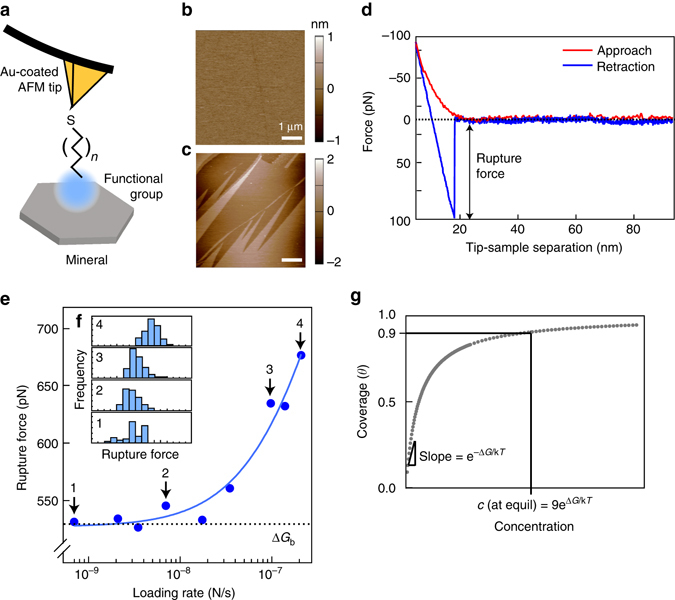
Dynamic force spectroscopy measurements at the organo–mineral interface. **a** Functional groups were covalently bound to gold-coated atomic force microscopy (AFM) tips and were used to probe mineral surfaces. Topographical AFM images of **b** the (001) surface of muscovite mica and **c** the (010) face of goethite (*bottom*) used in DFS experiments. *Scale bars*, 1 μm. **d** A representative force-distance curve and **e** a plot of mean rupture force versus loading rate showing increasing rupture force with increasing loading rate. The *solid line* represents a fit to the data using a theoretical model that accounts for both equilibrium and kinetic regimes of adsorption and forced desorption (See Eqs. 1–3, Methods section). Histograms (**f**, *inset*) of the force curves from individual points (1)–(4) show the trend of increasing rupture force with increasing loading rate. **g** To relate values of Δ*G* to typical measurements seen in adsorption experiments such as surface coverage, their relationship at equilibrium is shown in a model desorption isotherm. In the example, at a surface coverage of 90%, the value of Δ*G* is related through a simple exponential

To perform DFS measurements, we chose a model system of organic functional groups and minerals relevant to soils to directly measure the strength of binding at the organic–mineral interface while varying local environmental conditions. Organic functional groups with alkyl linkers were covalently bonded to gold-coated AFM tips as self-assembled monolayers and allowed to bind to one of two different mineral types: a phyllosilicate (muscovite mica) or an Fe (oxy)hydroxide (goethite) (Fig. 1, Fig. 2b, c). These minerals were chosen as models, because both permanently charged phyllosilicates and Fe oxides are ubiquitous in many soils and have been shown to have a high affinity for SOM^19, 20^. The functional groups COO^−^, PO_3_^−^, and NH_3_^+^ were chosen, as they are representative of chemistries found in SOM (Fig. 1). A representative force curve is shown in Fig. 2d, where a distinct rupture event is observed and can be used to obtain the rupture force. Since the binding can be described by a probability distribution for each ligand–mineral pair, hundreds of individual force curves were collected to constrain the average value (Fig. 2f﻿). As expected from the theory of DFS^14, 15^, the force required to rupture a bond increases with increasing pulling velocity (loading rate), as shown by a representative data set for interactions between a methyl group and mica (Fig. 2e). By fitting the data to extract the rupture force at zero loading rate, the quasi-static work of bond breaking, and hence the equilibrium free energy of binding Δ*G*_b_, is determined. To relate this value of Δ*G*_b_ to binding isotherms for organic matter and mineral surfaces that one would acquire in an adsorption/desorption experiment, one can convert Δ*G* to surface coverage, a commonly reported parameter in soil science (Fig. 2g).

### Quantifying binding of functional groups to model minerals

**Fig. 3:**
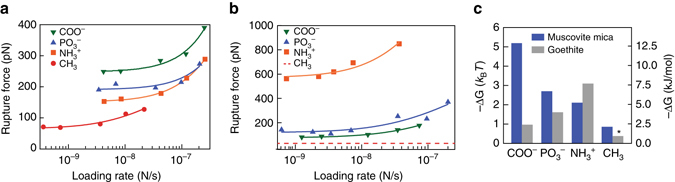
Chemistry of model soil organic matter affects binding strengths to different minerals. Experiments were performed at pH 6 in 10 mM NaCl. **a**, **b** Mean rupture forces for interactions between self-assembled monolayers with various functional groups and **a** muscovite mica or **b** goethite. **c** Experimentally derived values for the binding free energy (Δ*G*_b_) from fitted dynamic force spectroscopy data. Values for fitting parameters can be found in Supplementary Table 1. *Asterisk*: The value for the methyl group interacting with goethite is approximate, as the force was close to the detection limit of the experiment

SOM chemistry, soil mineralogy, and local environmental factors are thought to be key players in driving organic–mineral interactions. To address the first two variables, we evaluated the binding strength of various individual chemical functional groups on muscovite mica (Fig. 3a) and goethite (Fig. 3b) at pH = 6 in 10 mM NaCl. We investigated pH = 6 because it lies within the range observed for most soils that support growth of crops and plants, where organic matter content and accumulation are high. Additionally, an ionic strength of 10 mM is representative for a variety of soils^21^. The inert CH_3_ functional group served as a control and demonstrated minimal binding to both the mica (Δ*G*_b_ = 0.88 *k*_B_ *T*) and goethite surfaces (Δ*G*_b_ < 0.5 *k*_B_ *T*). In contrast, the strongest binding pair — COO^−^ on mica — exhibited a binding energy roughly an order of magnitude greater (Δ*G*_b_ = 5.8 *k*_B_ *T*) than the methyl control. When probing the role of chemistry, we observed the following trend of binding strengths on mica: COO^−^ > PO_3_^−^ > NH_3_^+^ > CH_3_, whereas a different trend was found on goethite: NH_3_^+^ > PO_3_^−^ > COO^−^ > CH_3_ (Fig. 3c and Supplementary Table 1). According to previously measured values for bond energies (Supplementary Fig. 3), the values for all of the bonds in our experiments are representative of non-covalent binding including hydrophobic and van der Waals interactions^4^. The results also show that below a loading rate of 10^−8^ N/s the near-equilibrium regime is reached for all organic–mineral pairs, as variation of the rupture force is minimal. Consequently, measurements of rupture force below this loading rate provide an approximate measure of the relative binding free energy.

At pH = 6, the mica basal surface will carry an overall negative charge due to isomorphic substitutions^22^, while the goethite will be below the point of zero charge and bear a partial positive charge (FeOH_2_^+^ groups dominate over FeO^−^ groups)^23^. Thus, the results in Fig. 3 display an overall trend where ligands and mineral surfaces with similar charge bind more strongly than oppositely charged pairs. For binding at the surfaces of phyllosilicates like mica, this result is understandable due to the discrete nature of the lattice, which allows organic molecules to bind with phyllosilicates through the cation sites that reside on the plane of oxygen atoms belonging to the tetrahedral silica sheet, despite the overall negative surface charge. While the latter would adversely impact the kinetics of binding for free ligands, it does not determine their binding energy (or coverage). Moreover, the nature of the DFS experiment, in which molecules are forced to the surface, ensures that the equilibrium bound state is sampled regardless of the kinetic barrier to adsorption that a net surface charge may create. Overall, the DFS results are consistent with the general conclusion that counterions (such as sodium, calcium, or carbonate) play a fundamental role in organic–mineral binding, particularly in moist environments where dissolved organic matter is expected to contact mineral surfaces.

Direct comparison between these DFS results and the results of bulk adsorption experiments on organic–mineral combinations^24, 25^ is challenging because very few techniques are capable of quantifying adsorption of a ligand to a single crystal face. For clay minerals such as goethite and mica, the relative areas of various crystal planes and edge sites can vary, altering the reactivity deduced from bulk samples^26, 27^. Yeasmin et al.^24^ combined FTIR with batch adsorption measurements comparing both amine- and carboxylic acid-bearing ligands on permanently charged phyllosilicates and observed that amines bound more strongly than carboxylic acids, likely due to interactions with variable charge edge sites. Our DFS results show a different trend compared to bulk measurements, however, the measurements are face-specific and were collected on the basal plane. Given that the charge state of the edge sites of phyllosilicates are pH-dependent, these sites are thought to be important for sorption of carboxylic acid-bearing ligands. Previous AFM-based force measurements have demonstrated that face-specific measurements of the edge and basal planes of mica have drastically different surface charge potentials^22^. Therefore, combining the DFS measurements of binding to the basal surface with studies demonstrating the preference of organic molecules for edge sites provides a more complete and mechanistic view of organic–mineral binding^28, 29^.

Comparing the existing literature with DFS results on goethite is also challenging. In particular, DFS shows relatively weak binding of carboxylic acid to the goethite (Fig. 3b, c) and FTIR spectroscopy shows that monocarboxylates bind with goethite through a weakly coordinated solvent-surface hydration-separated ion pair at circumneutral pH^30, 31^. In experiments using a batch adsorption approach, molecules containing multiple functional groups demonstrated a preference for phosphonic acid groups over amine or carboxylic acid groups^32^. While DFS did not demonstrate a preference of phosphoric groups over amine groups, physical measurements using CFM found positive correlations between nitrogen-containing aliphatic molecules and (oxy)hydroxide minerals^33^. This is consistent with the DFS results where stronger binding of the amine functional group compared to the carboxylic acid was observed at pH = 6 in 10 mM NaCl (Fig. 3c; Supplementary Table 2). Moreover, the (010) face is the preferred cleavage plane, and thus a low energy face of goethite, which may explain some of the discrepancy between FTIR experiments and DFS and CFM, as FTIR experiments probe all surfaces of the goethite crystals.

### The role of environmental factors in organic–mineral binding

**Fig. 4:**
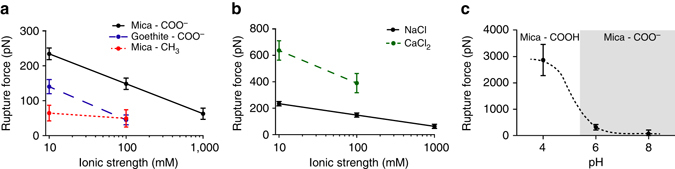
Values for the strength of binding as a function of local environmental conditions. Organic–mineral interactions are affected by ionic strength, ion composition, and pH. **a** Ionic strength was varied using NaCl electrolyte at pH = 6. Interactions between COO^−^ and either mica or goethite were investigated. The CH_3_ functional group was used as a control interaction on mica. **b** The effects of monovalent (NaCl) and divalent (CaCl_2_) cations were compared using the carboxylic acid functional group and mica at pH 6 at varying ionic strengths. **c** The effect of binding between carboxylic acid and mica was probed as a function of pH in 10 mM NaCl solution. For each condition, experiments were performed with the same AFM tip for reproducibility. Rupture forces were measured at loading rates of 3.5 × 10^−9^ N/s. Values are represented as mean ± 1 standard deviation

To understand the impact of environmental factors on these binding energies, we further evaluated the interaction of the organic–mineral pairs with changes in ionic strength (Fig. 4a), ion composition (Fig. 4b), and pH (Fig. 4c) in the near-equilibrium regime (i.e., low pulling rates). When varying ionic strength, we expected the functional group charge to be a factor, so we compared the behavior of an ionic carboxylic acid group to a non-polar methyl group at pH = 6 in NaCl. As expected, the methyl group showed no significant change in binding strength as a function of ionic strength. In contrast, the binding of the ionizable carboxylic acid group displayed a logarithmic dependence on ionic strength between 10 and 1000 mM, dropping by more than a factor of three on mica and exhibiting an even stronger reduction on goethite. A similar trend of decreasing binding force with increasing ionic strength was observed using CFM where the interactions between hydrophilic dissolved organic matter (identified to be rich in amino sugars, polysaccharides, and proteins) and mica were investigated^34^. Overall, ionic strength plays a key role in organic matter–mineral binding^35, 36^ and DFS suggests that polar interactions with charged organic ligands play a more important role at low ionic strength than at high ionic strength. At high ionic strength, charge screening and weakening of both electrostatic interactions and hydrogen bonding likely contribute to the reduced binding force.

To evaluate the effect of ion composition, the carboxylic acid–mica interaction was used to compare divalent calcium cations with monovalent sodium cations at various ionic strengths. Calcium ions demonstrated a threefold increase in binding strength compared to sodium and exhibited the same overall trend of reduced binding force with increasing ionic strength. These results are consistent with findings that calcium promotes cation bridging between surfaces and batch adsorption experiments demonstrate a strong correlation with increased adsorption of humic acid and extracellular polymeric substances to clays in the presence of divalent calcium ions^37, 38^. Additionally, calcium has been shown to be co-localized with carbon in organic–mineral associations from wetland soils, suggesting that calcium may play a significant role in aggregation^39^. The DFS experiments reported here allow us to quantify the relative effects of sodium and calcium cations on carboxylic acid binding to the basal plane of muscovite mica. The data corroborate previous literature results where higher sorption of organic matter is observed in the presence of calcium.

To investigate the effect of pH, we measured the binding energy between the carboxylic acid group and mica over the range of pH = 4–8, which spans the pKa reported for the ligand functionalized to the surface of the AFM tip (5.5 ± 0.5)^40^ and is a representative range for soil pH. When the pH was tested below the pKa (pH = 4), the binding force between carboxylic acid and mica increased by an order of magnitude (Fig. 4c) from 310 pN to nearly 3 nN, which corresponds to binding strengths previously reported for a covalent bond^41^. We hypothesize that at low pH, the mica surface is enriched with surface sites containing hydronium ions in which the hydrogens can be used to satisfy the protonated state of the carboxylic acid groups, resulting in strong covalent-like bonds at the surface and increased rupture forces at pH = 4 as compared to higher pH values. Experiments combining batch sorption with attenuated total reflectance (ATR)-FTIR and modeling have probed the bonding mechanisms of humic acid proxies containing carboxylic acid with illite and demonstrated a transition from a monodentate binding mechanism at neutral pH to a stronger bidentate binding complex at low pH^42^. This pH dependency has been reported for a number of organic–mineral systems, as stronger inner-sphere coordination generally occurs at low pH, while weaker outer-sphere complexation is observed at higher pH^20, 31, 43, 44^. Additionally, it is worthwhile to note that the force measurements were performed on the basal surface of mica, and while phyllosilicates are known to possess variable-charge surfaces at edge sites, this data shows that the basal surfaces are also capable of demonstrating pH-dependent behavior. Overall, the force spectroscopy results correlate with previous studies, suggesting that protonated carboxylic acid functional groups can bind strongly with negatively charged clay surfaces at low pH, which potentially represent interactions found in acidic subsoil horizons.

Additionally, we probed the role of carboxylic acid–goethite interactions with varying pH and observed an overall similar trend (Supplementary Fig. 2), however the magnitude of the forces was much smaller than those on mica. This result is somewhat surprising, as previous literature suggests that carboxylic acids can bind iron oxides strongly, particularly at low pH^45^. However, our experiment is probing a monocarboxylate, and thus weak monodentate binding, which binds through outer-sphere mechanisms. In particular, ATR-FTIR has demonstrated that monocarboxylates bind through a solvent-surface hydration-separated ion pair surface complex at near neutral pH and a surface hydration-shared ion pair complex at acidic pH^30^.

## Discussion

The observed trends with environmental conditions on both mica and goethite represent inherent properties of the system that should be considered when performing experiments at larger length scales. For example, when considering the impact of wetting and drying cycles on carbon dynamics, parameters such as ionic strength could play a direct role in adhesion of organic matter to mineral surfaces. This factor in organic–mineral binding is important to consider, as organic matter is transported through soils via water and water films and the interactions at mineral surfaces exhibit a temporal and spatial dependence. Parameters such as local environmental conditions, organic matter chemistry, soil structure/porosity, and mineral reactivity are key parameters that affect carbon decomposition rates^2^. DFS experiments are powerful because they can merge physical measurements with chemical analyses to make links between fundamental experiments with those performed on complex systems.

Of the experiments performed in the soil science community, DFS is most similar to CFM, which has been used previously to characterize binding strengths between natural or dissolved organic matter and Fe (oxy)hydroxide or mica minerals^33, 34, 46^. DFS is unique from CFM because it captures bond rupture over multiple loading rates and provides a dynamic viewpoint. While CFM does not provide the details of the bond lifetime or binding free energy, it can provide a magnitude for bond rupture forces and can be compared with DFS data to validate overall trends in binding. To connect the quantitative data obtained using DFS with techniques established in the soil science community, we chose to compare our data with batch adsorption/desorption measurements and CFM (Supplementary Table 3). In general, DFS shows similar trends as CFM. However, binding free energies calculated from DFS are lower than what is measured by batch adsorption experiments, likely because, for both mica and goethite, the DFS measurements were obtained on a single crystal face, which was flat and the low energy face and preferred cleavage plane.

The comparisons between batch adsorption experiments and DFS measurements highlights the fact that, in a number of instances, trends seen in the DFS data differ from those of previous bulk studies. In each case, our hypothesis is that the differences arise from the distinct nature of the surfaces probed in the two types of measurements. The literature offers strong evidence that large differences in face-specific ligand-surface binding should be expected. As noted above, surface charge potential measurements on muscovite demonstrated the pH sensitivity of edge sites and insensitivity of basal sites^22^. In addition, AFM force measurements of biologically relevant functional groups at different crystal faces of calcium oxalate crystals revealed significant differences in adhesion strength believed to impact the aggregation behavior^47^. While testing the above hypothesis is not possible with quasi-two dimensional minerals like mica and goethite, future studies of soil-relevant minerals with multiple facet types (e.g., quartz or hematite) could provide insight into face specificity of organic binding.

DFS has the potential to probe a variety of mineral–mineral, organic–mineral and organic–organic bonds relevant to soil science. While the experiments in this manuscript were meant as a proof of concept with pristine, flat mineral surfaces, nothing prevents DFS from being performed on heterogeneous surfaces. The number of measurements that must be performed would have to be large enough to average the binding forces over all surfaces to determine the binding energies of mineral–organic bonds. If one were to explore performing DFS on a real soil mineral surface, there are some limitations to DFS, but more complex systems could be imagined. In particular, a flat region (~100 nm^2^) would be preferred to prevent interactions from a combination of both edge and basal sites. Freshly cleaving the mineral is not necessary; however, natural adsorption of adventitious carbon to surfaces exposed to air over time could contribute to the final measurements. The localized nature of DFS measurements would enable researchers to build face-specific knowledge about organic–mineral interactions despite sample heterogeneity.

DFS is uniquely suited to quantifying the binding strength of biomolecules at the organic–organic or organic–mineral interface. Ex﻿periments could also be performed during biological activity, for example, in the presence of enzymes and/or live microbes, to obtain information about what stage of enzymatic secretion produces organic matter that is most reactive to mineral surfaces. Determination of the exact nature of the chemical bond would be challenging, but could potentially be addressed using simultaneous FTIR or Raman spectroscopy, including the use of tip enhancement^48, 49^.

Additionally, DFS can provide insight into current theories about how SOM interacts with mineral surfaces. For example, organic species in soils and marine sediments are thought to associate with mineral particles at distinct reactive sites and are distributed heterogeneously in multilayers^3, 50, 51^. The experiments presented here address the binding forces specifically at the organic–mineral interface and correspond to that of an ideal monolayer. While there is significant evidence suggesting that organics do not adsorb as monolayers, this initial measurement provides a stepping stone for evaluating multilayer interactions by providing information about forces present in the contact zone immediately adjacent to the mineral surface. Subsequent experiments to probe the multilayer model would involve molecular scale measurements of organic–organic interactions on mineral surfaces. Local environmental conditions that are varied in the experiments here would likely play a significant role, as long range interactions can extend for tens of nanometers from the mineral surface. Combining information about organic–mineral and organic–organic interactions could provide valuable insight into the binding forces that dominate in organo–mineral assemblages.

The approach to obtaining direct and quantitative treatment of the organic–mineral interface presented here could potentially provide fundamental information for next-generation land-carbon models where mineral-bound C is an important control on carbon persistence. These models are at the cutting edge of our current understanding of the terrestrial C cycle and provide a mathematical framework to describe SOM pools and their fluxes^52, 53^. Thus far, models have not yet incorporated parameters that consider direct measurements of carbon sorption and desorption under dynamic conditions at the mineral length scale. Additionally, the contribution of reactive mineral surfaces is often simplified by considering Langmuir adsorption kinetics, which may not provide an accurate view of mineral-bound C^54, 55^. Extending the measurements of binding free energy described here from simple functional groups to more complex, realistic biomolecules and systems in which biological activity is occurring can thus provide a significant advance in understanding soil C dynamics.

By systematically varying organic functional groups, our findings demonstrate that chemical structure plays a key role in binding strength between model organics and minerals, however, differences in local environmental conditions such as pH and ionic strength produce the most drastic differences in binding. With the current pace of climate change and the expected increases in the frequency of flooding and drought, it is crucial that we begin to understand how local, nano, and micron scale environmental conditions affect organic–mineral interactions and hence the stability of SOM. The approach used here demonstrates that directly measuring binding energies can provide an initial molecular basis for understanding organic–mineral interactions, potentially providing numerical values that can improve predictions of SOM persistence.

## Methods

### Materials

Muscovite mica (Ted Pella) and goethite (Cornwall, UK) were used as received, and both minerals were freshly cleaved along (001) or (010) cleavage planes, respectively, prior to experiments. Sodium chloride (99.99%), calcium chloride (99.99%), sodium hydroxide, and hydrochloric acid (Sigma-Aldrich) were used as received. Molecules for tip functionalization of self assembled monolayers of 11-amino-1-undecanethiol hydrochloride (99%), 11-mercaptoundecylphosphoric acid (95%), 12-mercaptododecanoic acid (96%), 1,11-undecanethiol (99%) (Sigma-Aldrich) were used as received.

Solutions with varying pH were adjusted using NaOH and HCl, and all solutions were filtered through a 0.22 μm filter prior to performing experiments.

### Dynamic force spectroscopy

AFM tips (SNL-10, Bruker) with a silicon tip and silicon nitride cantilever were coated on their front side with a 5 nm titanium adhesion layer and 20 nm gold coating (Bruker). SNL-10 or OBL-10 (Bruker) tips were cleaned under oxygen/argon plasma for a minimum of 5 min, rinsed in ethanol and fixed in a glass tube. The tip was then immersed for a minimum of 12 h in a 1 mM solution of the alkane thiol in ethanol (Methods section for details). The tips containing 12-mercaptododecanoic acid were functionalized in a 95 : 5 ethanol : acetic acid mixture. Following functionalization, tips were incubated in ethanol and rinsed with buffer before performing DFS measurements.

DFS measurements were performed at 25 °C using a Cypher ES Environmental AFM. Spring constants of each cantilever were measured using the thermal calibration method^56^. A minimum of five loading rates were chosen for each DFS measurement ranging from 10 to 3000 nm/s, and all force measurements were probed with a constant approach velocity of 100 nm/s and a dwell time both at the surface and away from the surface for 1 s with a 1-2 nm deflection trigger point. A minimum of 50 force curves were collected at each retraction velocity across numerous points on the sample surface to account for local heterogeneities, resulting in a minimum of 250 data points per sample.

Data was analyzed using the multiple bond theory developed by Friddle et al.^14^ where the energy profile at both near-equilibrium and far-from-equilibrium regimes are explored. Rupture forces vary as the logarithm of the loading rate and when evaluating both the physical distance between the unbound and bound states (*x*_t_) and extrapolating the data to force that is representative at a pulling rate of zero (*f*_eq_), which represents a quasi-equilibrium case where the work required to break a bond in the absence of pulling, the free energy of binding (Δ*G*_b_) can be evaluated. The mean rupture forces were fit to the following equation to determine experimental values for the equilibrium force *f*_eq_, distance between transition states *x*_t_, and intrinsic unbinding rate *k*_u_:

1 $${\left\langle f \right\rangle _N} \cong {f_{{\rm{eq}}}} + N{f_{\rm{\beta }}}{{\rm{e}}^{\frac{N}{{R\left( {\frac{{{f_{{\rm{eq}}}}}}{N}} \right)}}}}{{\rm{E}}_{\rm{1}}}\frac{N}{{R\left( {\frac{{{f_{{\rm{eq}}}}}}{N}} \right)}}$$

2 $$R\left( {\frac{{{f_{{\rm{eq}}}}}}{N}} \right) = \frac{r}{{{k_{\rm{u}}}\left( {\frac{{{f_{{\rm{eq}}}}}}{N}} \right)f{\rm{\beta }}}},{{\rm{E}}_1}\left( Z \right) = \mathop {\int}\limits_{\rm{z}}^\infty {\frac{{{{\rm{e}}^{{\rm{ - }}s}}}}{S}ds} $$

where *f*_β =_ *k*_B_ *T*/*x*_t_, *r* is the loading rate, and *N* is the number of bonds. The binding free energy of the bound state can then be evaluated from:

3 $$\Delta {G_{\rm{b}}} = {k_{\rm{B}}}T{\rm{ln}}\frac{{{f_{{\rm{eq}}}}{x_{\rm{t}}}}}{{{k_{\rm{B}}}}} + {f_{{\rm{eq}}}}{x_{\rm{t}}} + {k_{\rm{B}}}T$$

### Atomic force microscopy

Mica samples were freshly cleaved prior to imaging. Goethite samples were anchored to a mica substrate with epoxy, cleaned thoroughly with 0.5 M NaOH and 0.5 M HCl, then freshly cleaved using a razor blade along the (010) cleavage plane. Images were acquired at 25 °C using a Cypher ES Environmental AFM. Bruker Sharp Nitride Lever (SNL-10) tips with a nominal spring constant of 0.06 N/m were used in tapping mode in a buffer of 10 mM NaCl at ambient pH (pH ~ 6). Setpoints ranged from 200 to 500 mV at a scan rate of 1–1.5 Hz.

### X-ray diffraction

The surface plane of the freshly cleaved goethite crystals was confirmed as (010), indexed to the *Pbnm* space group with *a* = 4.608, *b* = 9.956, *c* = 3.0215, using a Rigaku Rapid II microbeam diffractometer equipped with a rotating Cr anode (*λ* = 2.2910 Å). The sample was positioned with its surface inclined at 45° vertically and 25° horizontally to the incident beam, and a series of two-dimensional diffraction patterns were recorded with sample rotated at different angles about an axis normal to its surface. Pole figures showing the intensity of the (130) and (021) Bragg peaks constructed from these data are shown in Supplementary Fig. 1 and show the symmetry and expected inclination (ca. 36° and 59°, respectively) to the (010) sample surface.

### Data availability

The data that support the findings in this manuscript are available from the corresponding author upon request.

## Electronic supplementary material

Supplementary Information
