# Author Correction: Developing a molecular picture of soil organic matter–mineral interactions by quantifying organo–mineral binding

**DOI:** 10.1038/s41467-017-02125-8

**Published:** 2017-12-05

**Authors:** C. J. Newcomb, N. P. Qafoku, J. W. Grate, V. L. Bailey, J. J. De Yoreo

**Affiliations:** 1 Pacific Northwest National Laboratory, 902 Battelle Blvd, Richland, WA 99354 USA; 2 Department of Materials Science and Engineering, University of Washington, 302 Roberts Hall, Seattle, WA 98195 USA

*Nature Communications* **8**:396 doi:10.1038/s41467-017-00407-9; Article published online: 30 August 2017

In the original version of this Article, the Acknowledgements section omitted the Department of Energy-funded Environmental and Molecular Sciences Laboratory in which the XRD measurements were performed. This error has now been corrected in both the PDF and HTML versions of the Article.

