# Supplementary material for: Developing a molecular picture of soil organic matter–mineral interactions by quantifying organo–mineral binding
Source: Nat Commun. 2017 Aug 30;8:396. doi: 10.1038/s41467-017-00407-9 (PMC5577185; doi:10.1038/s41467-017-00407-9)
Supplement: Supplementary file 1 — Supplementary Information [file 41467_2017_407_MOESM1_ESM.pdf]

Description of Supplementary Files

File Name: Supplementary Information
Description: Supplementary Figures, Supplementary Tables and Supplementary References

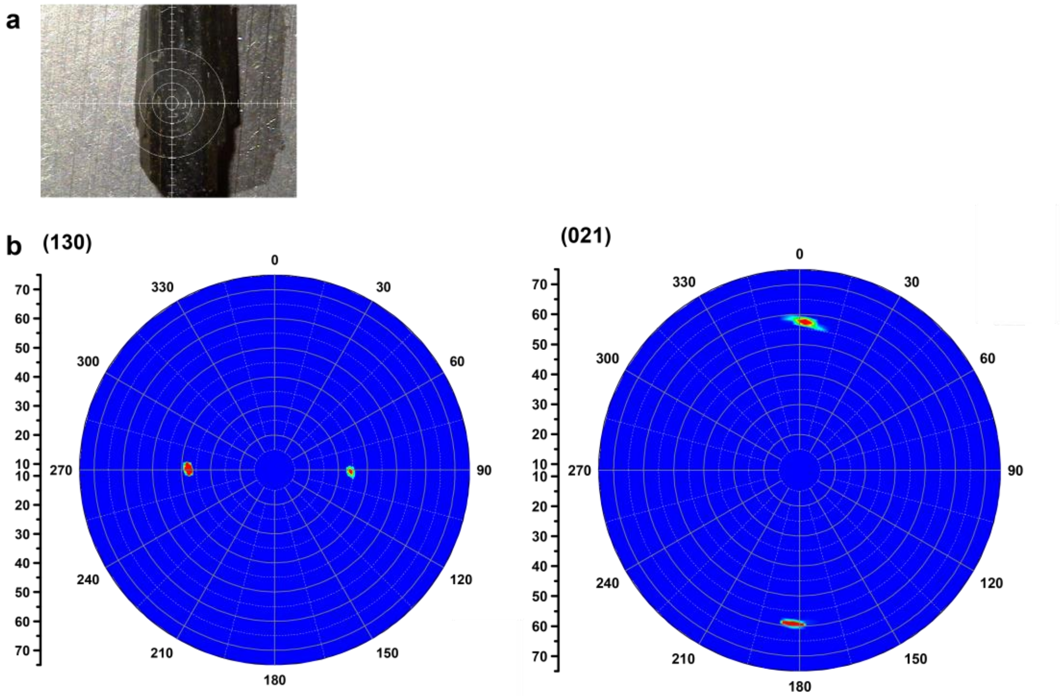

Supplementary Figure 1. (**a**) An image of the goethite crystal with the (010) surface cleaved face up. (**b**) Pole figures showing the intensity of selected Bragg peaks with respect to the sample surface. The (130) planes are tilted about 36° from the surface and the (021) Bragg peaks are tilted 58° from the surface, indicating that the (010) plane is normal the sample surface.

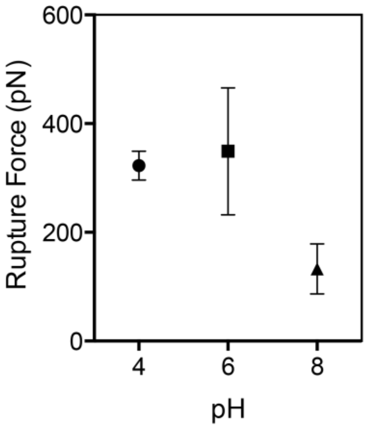

Supplementary Figure 2. The effect of binding between carboxylic acid and goethite with changes in pH. Experiments were performed in 10 mM NaCl. Rupture forces were measured at loading rates of 3.5×10^{-9} N/s. Values are represented as the mean ± one standard deviation.

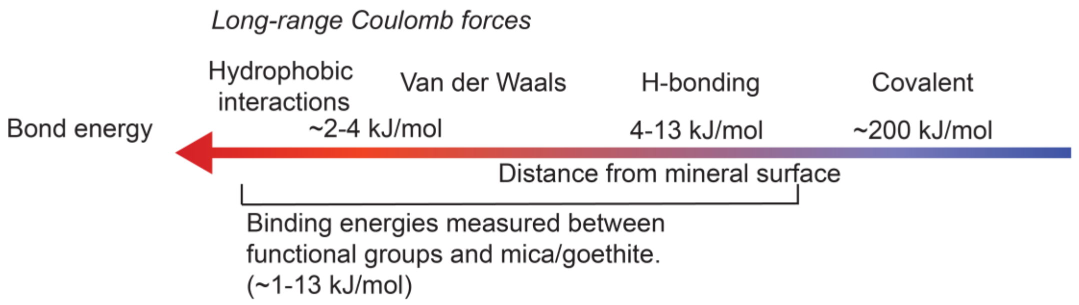

Supplementary Figure 3. A schematic representation showing typical values for binding energies as a function of distance from the surface and corresponding values measured in DFS between organic ligands and mineral surfaces. Distance between the bond ligand and mineral surface increases from right to left while bond energies increase from left to right.

Mineral: Muscovite mica		Fitting Parameters		
Organic ligand	$-\Delta G_{bu}$ (k_BT)	x_t (Å)	k_o (s^{-1})	f_{eq} (pN)
COO^-	5.78 ± 0.67	63.2 ± 1.4	39.4 ± 0.2	229 ± 41
NH_3^+	2.12 ± 0.5	30.1 ± 1.2	279 ± 65	146 ± 48
PO_3^-	2.71 ± 0.66	39.2 ± 2.9	356 ± 102	190 ± 10
CH_3	0.88 ± 0.30	72.1 ± 6.0	62.12 ± 1.71	65.7 ± 6

Mineral: Goethite		Fitting Parameters		
Organic ligand	$-\Delta G_{bu}$ (k_BT)	x_t (Å)	k_o (s^{-1})	f_{eq} (pN)
COO^-	0.98 ± 0.57	29.8 ± 26.9	229.9 ± 117	147.4 ± 69.1
NH_3^+	3.11 ± 0.17	12.9 ± 9.1	110.3 ± 16.7	557.4 ± 16.5
PO_3^-	1.63 ± 0.85	28.9 ± 9.7	103.61 ± 92	117.8 ± 26.3
CH_3	< 0.5			

Supplementary Table 1. Values for ΔG_{bu} and corresponding fitting parameters from dynamic force spectroscopy data (x_t: distance to transition between bound and unbound states, k_o: intrinsic unbinding rate of the complex, f_{eq}: equilibrium force). All values are represented as mean ± standard deviation.

Comparison of DFS Results with Bulk Experiments

Ligand	Mineral	Expt conditions	Methods	Ref	Binding value	Relevant DFS Experiment
$CH_3(NH_2)_nNH_2$<br><br>*n = 1*<br>*n = 3*<br>*n = 5*	Vermiculite	Measured values thought to represent intercalated material. Aqueous, 10mL, 30 mg vermiculite,	Batch adsorption, calorimetry	1	(kJ/mol)<br>ΔG = -30.51 ± 0.3<br>ΔG = -38.08 ± 0.1<br>ΔG = -30.09 ± 0.3	NH_2-muscovite mica<br><br>ΔG = -15 kJ/mol
Dicarboxylate ligand<br><br>Succinate (claimed monodentate binding mechanism)	Hematite	5 mM acetate, pH = 5, 2 g/L hematite, 25C	ATR-FTIR	2	Succinate: Langmuir binding constant K = 2700 ± 300 M^{-1}<br>ΔG ≈ -18 kJ/mol	COO^{-}goethite<br><br>ΔG = -2.5 kJ/mol

Supplementary Table 2. Comparison of DFS data to bulk adsorption/desorption/ATR-FTIR methods probing organic-mineral binding. Comparing the value for binding free energy of similar interactions using bulk methods and a similar DFS experiments show that DFS values are lower than those found using bulk methods.

Comparison of DFS Results with Chemical Force Microscopy

<table>
<tr><th>Ligand</th><th>Mineral</th><th>Experiment conditions</th><th>Ref</th></tr>
<tr><td>Natural organic matter from different sources</td><td>Mica</td><td>Ambient pH, range of ionic strength (0-100 mM NaCl)</td><td>3</td></tr>
<tr><td colspan="4">Comparison of Results
Ref (3): Hydrophilic DOM > methyl groups + polysaccharide-rich DOM > fulvic acid structure (aromatic) DOM
This paper: $COO^- > NH_3^+ > CH_3$ on Mica</td></tr>
<tr><td>Dissolved organic matter, IHSS Pahokee Peat</td><td>Iron (oxy)hydroxide</td><td>pH 4.65, 10 mM sodium acetate</td><td>4</td></tr>
<tr><td colspan="4">Comparison of Results:
Ref (4): Carboxyl containing aromatics and N-aliphatics > lignin/phenolic, non-N aliphatic
This paper: $NH_3^+ > COO^- > CH_3$ on goethite</td></tr>
</table>

Supplementary Table 3. Comparison of dynamic force spectroscopy data to chemical force microscopy data performed on environmental organic-mineral samples. The trends in chemistry were consistent between current literature and the fundamental studies performed in this manuscript.

Supplementary References

1. da Fonseca, M. G. *et al.* Vermiculite-aliphatic amine interactions at the solid/liquid interface: a thermodynamic approach. *J Therm Anal Calorim* **97,** 453–457 (2009).
2. Duckworth, O. W. & Martin, S. T. Surface complexation and dissolution of hematite by C1-C6 dicarboxylic acids at pH = 5.0. *Geochimica et Cosmochimica Acta* **65,** 4289–4301 (2001).
3. Aubry, C., Gutierrez, L. & Croue, J. P. Coating of AFM probes with aquatic humic and non-humic NOM to study their adhesion properties. *Water Research* **47,** 3109–3119 (2013).
4. Chassé, A. W. *et al.* Chemical Force Spectroscopy Evidence Supporting the Layer-by-Layer Model of Organic Matter Binding to Iron (oxy)Hydroxide Mineral Surfaces. *Environ. Sci. Technol.* **49,** 9733–9741 (2015).
